# Does community-based health insurance reduce disparities in modern health service utilization among households in Ethiopia? A community-based comparative cross-sectional study

**DOI:** 10.3389/fpubh.2022.1021660

**Published:** 2023-01-13

**Authors:** Edosa Tesfaye Geta, Abebe Wakjira Bidika, Belachew Etana

**Affiliations:** ^1^Department of Public Health, Institute of Health Science, Wollega University, Nekemte, Ethiopia; ^2^Gida Ayana Hospital, Nekemte, Oromia, Ethiopia

**Keywords:** CBHI, disparities, modern health services, health care utilization, Gida Ayana District, Ethiopia

## Abstract

**Background:**

Community-based health insurance (CBHI) is an emerging and promising concept to access affordable and effective healthcare by substantially pooling risks to improve health service utilization (HSU) and equity. While there have been improvements in healthcare coverage in Ethiopia, disparities in healthcare remain a challenge in the healthcare system. Hence, the study aimed to assess the effects of CBHI on the reduction of disparities in modern health service (MHS) utilization among households.

**Methods:**

A community-based comparative cross-sectional study was conducted between 1 February and 30 April 2022 among households in the Gida Ayana district, Ethiopia. The sample size of 356 was determined using the double population proportion formula, and participants were selected using multistage sampling. Data were entered into EpiData 4.6 and exported to SPSS 25 for analysis.

**Results:**

Among 356 households, 321 (90.2%) reported that at least one member of their family fell ill in the previous 6 months; 153 (47.7%) and 168 (52.3%) households were among the insured and uninsured, respectively. Only 207 [64.5, 95% confidence interval (CI) = 59.0–69.7%] of them utilized health services. The level of MHS was 122 (79.7, 95% CI = 75.5–85.8%) and 85 (50.6, 95% CI = 42.8–58.4%) among insured and uninsured, respectively. Insured households were four times more likely to utilize MHS compared to uninsured households [adjusted odds ratio (AOR) = 4.27, 95% CI = 2.36–7.71]. Despite the households being insured, significant disparities in MHS utilization were observed across the place of residence (AOR = 14.98, 95% CI = 5.12–43.82) and education level (AOR = 0.20, 95% CI = 0.05–0.83).

**Conclusion:**

Overall, the CBHI scheme significantly improved the level of MHS and reduced disparities in utilization across wealth status and family size differences. However, despite households being insured, significant disparities in the odds of MHS utilization were observed across the place of residence and education level. Hence, strengthening the CBHI scheme and focusing on the place of residence and the education level of households are recommended to improve MHS utilization and reduce its disparities.

## Introduction

The goal of universal health coverage (UHC) is to ensure that all people have access to affordable and quality health services, regardless of their economic status, gender, or other characteristics. However, disparities in UHC do exist both between and within countries (1).

Healthcare utilization is the quantification or description of the use of services by people to prevent and cure health problems, promote the maintenance of health and wellbeing, or obtain information about the health status and prognosis of a person (2).

In healthcare disparities (HCD), certain groups have disproportionately poor access to affordable care, including a lack of insurance or the means to afford insurance or care, as well as poor access to providers or transportation, and these populations also experience disparities in treatment, quality of care, and health outcomes (3). Health is unevenly distributed across socioeconomic status. People of lower income, education, or occupational status experience worse health and die earlier than their better-off counterparts (4).

Community-based health insurance is a prepayment form of healthcare financing that is usually organized at the community level and has the outstanding feature of being run as a not-for-profit scheme. It targets the informal sector and applies the basic principles of risk sharing and member participation in the management of the schemes. This ensures that the healthy and wealthy cross-subsidize the costs of health services for the ill and low-income people, and its aim is also to improve health service utilization (5, 6).

The World Health Organization (WHO) considers health insurance as a promising means for achieving universal health coverage (UHC), and governments of different countries of the world are strengthening the scheme to pursue financing systems for health services that avoid two important risks, namely, the risk of catastrophic expenditure (leading to impoverishment), caused by the need for large out-of-pocket payments for medical services when a person falls ill, and the risk that even modest user fees may dampen demand and create barriers to access for the poorest members of the population (7).

Despite continued global agreement on the need to strengthen national health financing systems to develop sustainable and comprehensive policies, health financing in low- and middle-income countries (LMIC) and individuals' access to essential health services depend on out-of-pocket expenditure, which is a major problem in LMICs for the provision and utilization of health services. According to the WHO, out-of-pocket expenditures of 15%−20% of total health expenditures or 40% of household net income for subsistence needs can lead to financial catastrophe (5, 8).

Globally, every year, approximately 44 million households or more than 150 million individuals face catastrophic expenditures (5). In sub-Saharan Africa (SSA), out-of-pocket expenditures constitute ~40% of total health expenditures (9).

There are still 1.3 billion people in the world with very low incomes who lack access to effective and affordable health services (1). When people with low incomes and no financial risk protection fall ill, they face a dilemma: they may use health services (if available) and suffer impoverishment from paying for healthcare, or they may forego services, remain ill, and risk being unable to work or function (5, 10).

Low- and middle-income countries suffer from the catastrophic financial burden of out-of-pocket payments (OOP), which accounts for 30%−85% of total healthcare spending (9). In sub-Saharan Africa (SSA), out-of-pocket expenditures constitute ~40% of total health expenditures, imposing huge financial burdens and limiting access to and utilization of health services (10).

The World Health Organization recommends around three to four outpatient department visits per person per year (11), and the health service utilization rate in SSA in particular is very low, ranging from 0.2 to 2 visits annually (12).

Poor healthcare-seeking behavior and utilization are major factors contributing to increased morbidity and mortality among the population in LMICs. In SSA countries, the percentage of people seeking healthcare is low, as reported in Mongolia (44.1%), Congo (54.6%), and Ethiopia (38.7%) (13).

Ethiopia is the second largest country in Africa in terms of population size. However, the country ranks low in access to modern healthcare services compared to African countries (14), and 35.5% of healthcare expenditures in the country are generated from households as a means of OOP, which hinders modern health services seeking and utilization (10).

In 2015, the average outpatient department (OPD) visit rate in Ethiopia was 0.48 visits per person per year; however, the target was two visits per person per year by 2020, which is low compared to other world countries where the global outpatient age-standardized utilization rate was 5.4 visits per individual per year (15, 16).

Community-based health insurance was launched in 2011 for the rural population and the informal sector in urban areas to reduce household vulnerability to OOP healthcare expenditure, increase the quality of services, and increase health service utilization (10). It is recognized as a powerful method to achieve UHC with adequate financial protection for all against healthcare costs and increasing health service utilization. The government of Ethiopia aimed to achieve UHC for its citizens by the end of 2030. To meet this, the CBHI scheme in all rural parts of the country and the informal sectors is considered one of the strategies (15).

However, the effect of the CBHI scheme on modern health service utilization was not well estimated in the country. Hence, the study aimed to estimate the effect of CBHI on the modern health service utilization among households in the Gida Ayana district, East Wollega Zone, Oromia regional state, Ethiopia.

## Methods and materials

### Study area and period

This study was conducted between 1 February 2022 and 30 April 2022 in the Gida Ayana district, which is located in the East Wollega Zone, Oromia Regional State, Ethiopia. It is located in the Oromia regional state to the west, at (90°52°N) and (42° 37°E), 430 km from Addis Ababa, the capital city of the country. The total catchment area of the district was about 183,063 m^2^. Its total population was estimated to be 135,980, of which 69,350 were women and 66,630 were men. The district had one general hospital, five health centers, 29 health posts, and 26 private clinics. According to the District Health Office 2019/2022 report, in the district, there were 30,357 households, and 14,670 (48.3%) of them were CBHI members.

### Study design and population

A community-based comparative cross-sectional study was used. All households in the Gida Ayana district were considered the source population, and systematically selected households were considered the study population, whereas the selected head of each household was considered the study unit.

### Eligibility criteria

Household heads or family representatives who were ≥18 years of age and who had resided in the area for more than 6 months were included in the study, whereas households that were unavailable during the study period were excluded from the study.

### Sample size determination and sampling techniques

The sample size was determined using the double population proportion formula (17). The healthcare utilization among insured households was 50.5% and among uninsured households was 29.3% (18) with a 95% confidence level and a power of 80%. Thus,


$$n\;=\;2{\left(Z_{\alpha /2}\pm Z_{\beta }\right)}^{2}\;*\;(p_{1}\left(1-p_{1}\right)+p_{2}\left(1-p_{2}\right))/(p_{1}-p_{2)}{}^{2},$$

where *Z*__α_/2_ = critical value at 95% CI which equals 1.96 (*Z* value at alpha = 0.05) and *Z*_β_ = the power to detect a difference in the two proportions, which is 0.84 (at β = 0.2), whereas *p*_1_ = proportion of healthcare utilization among insured households and *p*_2_ = proportion of healthcare utilization among uninsured households.

Their values were then replaced in the formula as follows:


$$\begin{array}{l} n\;=\;2(1.96+0.84)2*(0.505(1-0.505)\\ \;+0.293(1-0.293))/\;(0.505-0.293)2 \end{array}$$

*n* = 80.32, which is equal to 81, and then, by multiplying by 2, *n* = 160.

The sample size was 160^*^2 = 324 when the design effect was considered. Finally, by adding the 10% non-response rate, the final sample size to be included in the study was 356 (178 insured households and 178 non-insured insured households).

All the lists of 28 kebeles in the district were taken from the administrative office of the district, out of which three kebeles were selected randomly using the lottery method. In the second stage, 22 zones based on their proportion to each kebele were randomly selected. The list of CBHI scheme member households was taken from the district community-based health insurance office, and the total number of non-CBHI households was calculated by subtracting CBHI scheme members from the total households in the selected zones.

Finally, the required sample size for both groups in each of the selected zones was determined using the population proportionate to the sample size (PPS), and systematic random sampling was used to select the study subjects for both the CBHI member and the comparative, non-member groups in each of the selected zones. Because of the difference in the number of households, “*K*” was calculated separately for each zone and each member (insured and uninsured) by dividing the total number of households in each zone (*S*) by the corresponding sample size (*s*). The number “*K*” obtained by dividing *S*/*s* was used to identify the interval among selected households from each zone.

**Figure 1:**
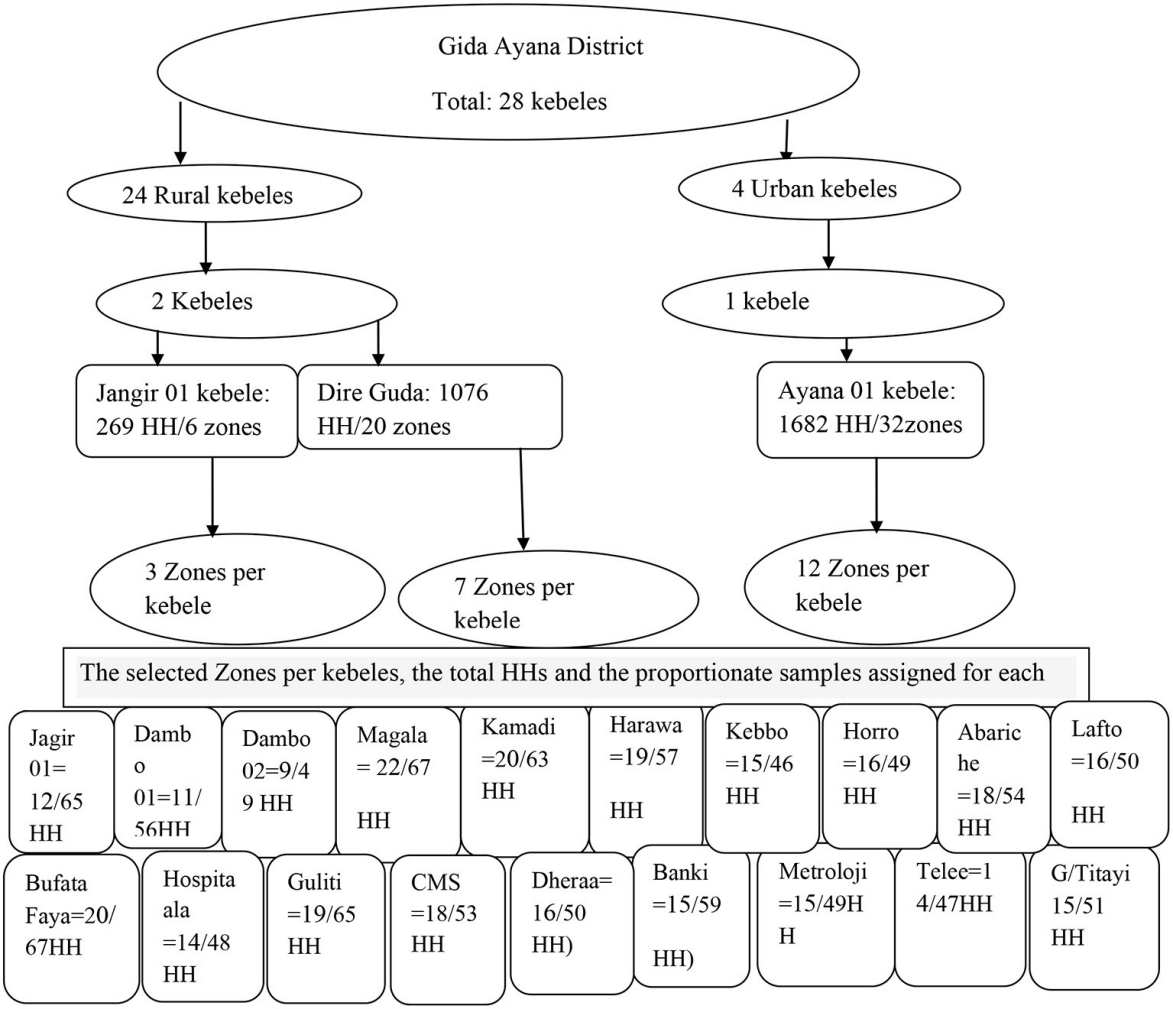
Sampling technique to select study households in Gida Ayana district, 2022.

Finally, since the sampling fraction was *K*, every *K*th household was included in the study, and to select the first household from 1 to *K*, the lottery method was used. In each household, one respondent was interviewed. If there was more than one eligible respondent in the compound, the head of the household was selected to be interviewed (Figure 1).

### Data collection tool and procedures

Semi-structured questionnaires were developed after reviewing different literature sources. All tools were prepared in English language and translated into Afan Oromo language for the interview. Data were collected through face-to-face interviews with household heads.

### Study variables

Modern health service utilization was measured as a dependent variable, and the independent variables were factors related to the household heads (age, sex, religion, marital status, occupation, education, family size, wealth status, and residence), CBHI scheme-related factors (insured household or uninsured household), and health facility-related factors (type of health facility and time to travel to the health facility).

### Operational definitions

#### Modern health services

Health services were provided by licensed health institutions, including public and private (hospitals, health centers, clinics, health posts, and private non-profit organizations).

#### Modern health service utilization

Utilization of health services was measured as the number of service utilizations (diagnosis or treatment) from modern health facilities made by at least one household member at least one time in the previous 6 months. It was a binary dichotomous variable measuring health service utilization, coded as 1 (visited a modern health facility) and 0 (did not visit a modern health facility) based on the question, “*Did you/your families visit modern health facilities for healthcare in the last 6 months*?”.

### Data quality management

To ensure the quality of research data, data collectors and supervisors received 2 days of training on the objective, methodology, sampling technique, ethical issues, data collection instrument, and data collection procedures. Data were collected by five experienced health professionals with a bachelor's degree and two supervisors with a master's degree in health. After discussion and a common understanding of the data collection tool, there was regular cross-checking by the data collectors for the completeness of the questionnaires, and the data collectors strictly followed the data collection procedure.

A pretest was done on 18 (5%) of the calculated sample size in another kebele of the Gida Ayana district. During the data collection period, close supervision and monitoring were done by the team to ensure the quality of the data. The completeness and consistency were checked in the field by the data collectors.

### Data analysis and presentation

Data were cleaned and entered into EpiData manager 4.6 version and exported to SPSS version 25 for analysis. Descriptive statistics were computed and presented using frequencies, proportions, summary statistics, graphs, and tables. Initially, a binary logistic regression analysis was computed to identify the significant effect of each independent variable on the utilization of health services, and then, to identify potential candidate variables at a *p*-value of < 0.25 for the final model, a multivariable logistic regression was used to determine the effect of CBHI membership and other factors on the probability of utilization of health services. The final *p*-value of < 0.05 was considered to declare the significant factors along with the odds ratio (OR) and the corresponding 95% CI.

## Results

### Sociodemographic and economic characteristics of study participants

A total of 356 heads of household participated in the study, with a response rate of 100%. The average age of the respondents was 38.21 ± 8.5 years. Heads of household were predominantly men (312, 87.6%) and married (333, 93.5%). Regarding the place of residence, the majority of them (230, 66.4%) were urban residents. The majority of the household heads (225, 63.2%) were farmers, and their wealth status was computed, showing that 277 (77.8%) of them were relatively poor. Regarding family size, the majority of the households (254, 71.3%) had a minimum of five family members, and 153 (43%) of the participants had no formal education (Table 1).

**Table 1:** Sociodemographic and economic characteristics among households in the Gida Ayana district, Oromia Regional State, Ethiopia, 2022.

**Variables** **(*n* = 321)**	**Response category**	**CBHI membership**	**Modern health services utilization**		
			**Yes (%)**	**No (%)**	**Total (%)**
Place of residence	Urban	Yes	89 (44.3)	5 (2.5)	94 (46.8)
		No	62 (30.8)	45 (22.4)	107 (53.2)
	Rural	Yes	33 (27.5)	26 (21.7)	59 (49.2)
		No	23 (19.2)	38 (31.7)	61 (50.8)
Sex	Men	Yes	100 (35.7)	31 (11.1)	131 (46.8)
		No	70 (25)	79 (28.2)	149 (53.2)
	Women	Yes	22 (53.7)	0 (0)	22 (53.7)
		No	15 (36.6)	4 (9.8)	19 (46.3)
Age	20–29	Yes	30 (47.6)	6 (9.5)	36 (57.1)
		No	16 (25.4)	11 (17.5)	27 (42.9)
	30–39	Yes	60 (45.1)	9 (6.8)	69 (51.9)
		No	32 (24.1)	32 (24.1)	64 (48.1)
	40–49	Yes	20 (23.3)	10 (11.6)	30 (34.9)
		No	28 (32.6)	28 (32.6)	56 (65.1)
	Above 50	Yes	12 (30.8)	6 (15.4)	18 (46.2)
		No	9 (23.1)	12 (30.8)	21 (53.80)
Family size	< 5	Yes	45 (52.3)	6 (7)	51 (59.3)
		No	27 (31.4)	8 (9.3)	35 (40.7)
	≥5	Yes	77 (32.8)	25 (10.6)	102 (43.4)
		No	58 (24.7)	75 (31.9)	133 (56.6)
Education	No formal education	Yes	44 (29.9)	19 (12.9)	63 (42.9)
		No	36 (24.5)	48 (32.7)	84 (57.1)
	Primary	Yes	21 (27.6)	7 (9.2)	28 (36.8)
		No	27 (35.5)	21 (27.6)	48 (63.2)
	Secondary and above	Yes	57 (58.2)	5 (5.1)	62 (63.3)
		No	22 (22.4)	14 (14.3)	36 (36.7)
Wealth status	Poor	Yes	97 (37.3)	26 (10)	123 (47.3)
		No	62 (23.8)	75 (28.8)	137 (52.7)
	Rich	Yes	25 (41)	5 (8.2)	30 (49.2)
		No	23 (37.7)	8 (13.1)	33 (50.8)
Distance from the health facility	< 1 h	Yes	76 (45)	2 (1.2)	78 (46.2)
		No	50 (29.6)	41 (24.3)	91 (53.8)
	≥1 h	Yes	46 (30.3)	29 (19.1)	75 (49.3)
		No	35 (23)	42 (27.6)	77 (50.7)

### Modern health service utilization

From a total of 356 heads of household who participated in the study, 321 (90.2, 95% CI = 86.6–93.3%) reported their perceived morbidity that at least one member of their family developed at least one recent illness episode in the previous 6 months. Among them, 153 (47.7%) and 168 (52.3%) were CBHI scheme members and non-members, respectively (Table 1).

**Figure 2:**
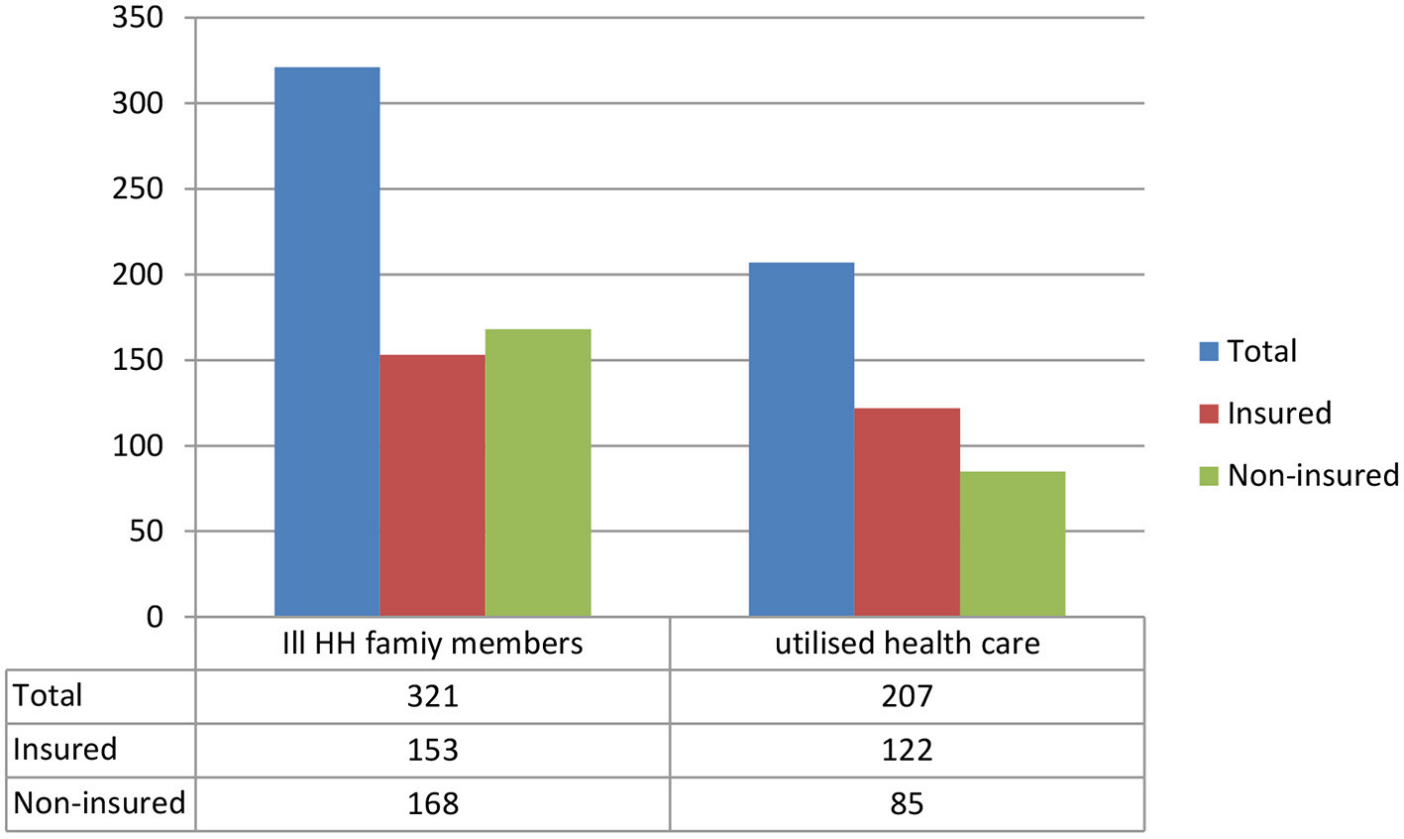
Household family members ill in the past 6 months and healthcare utilization among households in the Gida Ayana district, Oromia Regional State, Ethiopia, 2022.

The study determined the level of MHS utilization for perceived morbidity among insured and uninsured households. Out of 321 households whose at least one family member fell ill, only 207 (64.5, 95% CI = 59–69.7%) of them utilized MHS. This health service utilization was higher among insured households, which was 122 (79.7, 95% CI = 75.5–85.8%), whereas, among uninsured households, it was 85 (50.6, 95% CI = 42.8–58.4; Figure 2).

More than half of the households (85, 71.4%), with at least one family member who became ill and visited the modern health facilities within 1 day of the onset of the illness, were insured, whereas the majority of those ill individuals who visited the modern health facilities after 5 days from the onset of the illness (29, 70.7%) were from uninsured households (Table 2).

**Table 2:** Time taken in days to visit modern health facilities among households in the Gida Ayana district, Oromia Region, Ethiopia, 2022.

**Days to visit modern health facility after onset of the illness (*n* = 207)**	**CBHI status**		
	**Insured (%)**	**Uninsured (%)**	**Total (%)**
Within 1 day	85 (41.1)	34 (16.4)	119 (57.5)
2–4 days	25 (12.1)	22 (10.6)	47 (22.7)
5–7 days	7 (3.4)	20 (9.7)	27 (13)
8–10 days	5 (2.4)	8 (3.9)	13 (6.3)
11–14 days	0 (0)	0 (0)	0 (0)
After 15 days	0 (0)	1 (0.5)	1 (0.5)
Total	122 (58.9)	85 (41.1)	207 (100)

**Figure 3:**
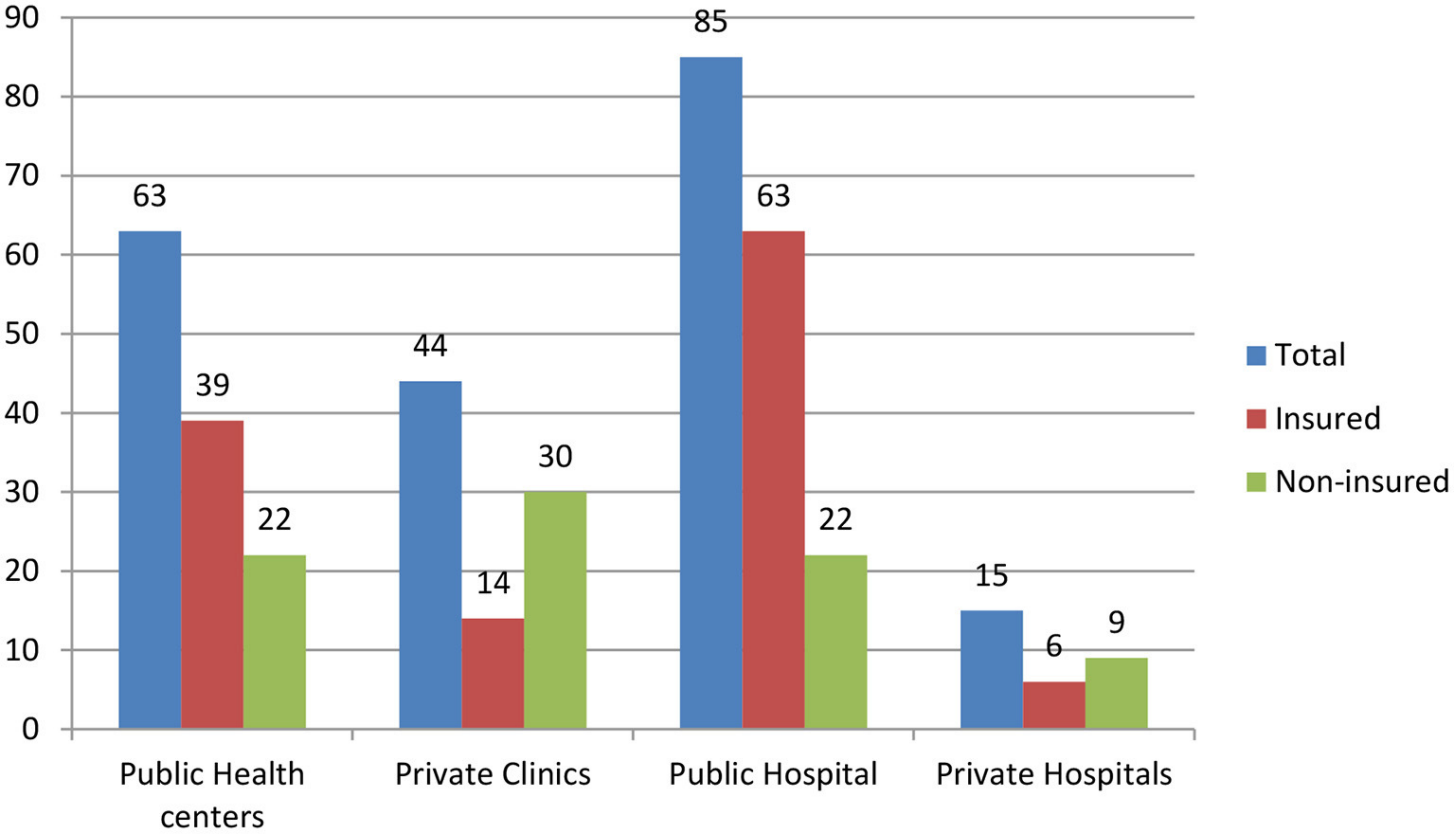
Type of modern health facilities visited by households (*n* = 207), in the Gida Ayana district, Oromia Regional State, 2022.

To utilize the MHS, the households visited different health facilities, including public and private health facilities (Figure 3).

Of the total individuals who visited private health facilities, the majority of them (39, 66.1%) were from uninsured households, whereas the majority (102, 68.9%) of the insured households visited public health facilities. More than half of the households (144, 54.1%) traveled to the nearest health facility for < 1 h to seek and utilize health services.

The number of visits per household was 2.96 ± 1.00 among the insured and 1.45 ± 0.50 among the non-insured. Being an insurance member among households, on average, increased the number of modern health facility visits by 1.5 compared to non-insured households, 95% CI = 1.51 (1.30–1.71), *t* = 12.818, *p* = 0.00 visits per household.

### Predictors of modern health service utilization

Controlling other explanatory variables, the study estimated the effect of health insurance on MHS utilization (Table 3). Accordingly, insured households were 4.3 times more likely to utilize MHS (AOR = 4.27, 95% CI = 2.36–7.71) compared to uninsured households, which significantly improved the utilization of healthcare among insured households.

**Table 3:** Predictors of modern health service utilization among households (*n* = 321) in the Gida Ayana district, Oromia Regional State, Ethiopia, 2022.

**Variables**	**Response category**	**Modern health service utilization**			**Odds ratio and 95% CI**	
**Yes (%)**		**No (%)**	**Total (%)**	***COR***	***AOR***
Place of residence	Urban	151 (47)	50 (15.6)	201 (62.6)	3.45 (2.14–5.58)	4.52 (1.61–12.73)^**^
	Rural	56 (17.4)	64 (19.9)	120 (37.4)	1	1
Age	20–29	46 (14.3)	17 (5.3)	63 (19.6)	2.32 (1.00–5.37)	1.29 (0.45–3.71)
	30–39	92 (44.4)	41 (36)	133 (41.4)	1.92 (0.93–3.98)	1.34 (0.57–3.17)
	40–49	48 (15)	38 (11.8)	86 (26.8)	1.08 (0.51–2.32)	1.48 (0.62–3.54)^**^
	50 above	21 (6.5)	18 (5.6)	39 (12.1)	1	1
Wealth status	Poor	159 (49.5)	101 (31.5)	260 (81)	0.43 (0.22–0.83)	0.33 (0.15–0.70)^**^
	Rich	48 (15)	13 (4)	61 (19)	1	1
Occupation	Farmer	125 (37.7)	81 (26.5)	206 (64.2)	0.48 (0.29–0.80)	0.79 (0.41–1.54)
	Merchant	86 (9)	29 (26.8)	115 (35.8)	1	1
Education level	No formal	80 (24.9)	67 (20.9)	147 (45.8)	0.29 (0.16–0.52)	0.34 (0.17–0.68)^**^
	Primary	48 (15)	28 (8.7)	76 (23.7)	0.41 (0.21–0.82)	0.56 (0.25–1.22)
	Secondary and above	79 (24.6)	19 (5.9)	98 (30.5)	1	1
Family size	< 5	72 (22.4)	14 (4.4)	86 (26.8)	3.81 (2.03–7.14)	2.52 (1.15–5.51)^*^
	≥5	135 (42.1)	100 (31.2)	235 (73.2)	1	1
Distance from HF	< 1 h	126 (39.3)	43 (13.4)	169 (52.6)	2.57 (1.61–4.11)	0.86 (0.30–2.44)
	≥1 h	81 (25.2)	71 (22.1)	152 (47.4)	1	1
CBHI status	Insured	122 (38)	31 (9.7)	153 (47.7)	3.84 (2.34–6.31)	4.27 (2.36–7.71)^***^
	Non-insured	85 (26.5)	83 (25.9)	168 (52.3)	1	1

^*^p-value < 0.05.

^**^p-value < 0.01.

^***^p-value < 0.001.

1, reference; CI, confidence interval, HF, health facility; COR, crude odds ratio; AOR, adjusted odds ratio.

The study also identified other variables that significantly affected the utilization of health services among households in addition to the CBHI scheme. Urban residents of households were 4.5 times more likely to utilize healthcare compared with rural residents (AOR = 4.52, 95% CI = 1.61–12.73).

Regarding the education level, household heads who had no formal education were 66% less likely to utilize modern health services (AOR = 0.34, 95% CI = 0.17–0.68) when compared to those household heads who attended secondary school and above. Relatively poor households were 67% less likely to utilize modern healthcare when compared to those that were relatively rich (AOR = 0.33, 95% CI = 0.15–0.70).

In addition, the family size was identified as a significant variable to affect the utilization of health services, and the study found that households with less than five family members were 2.5 times more likely to utilize health services (AOR = 2.52, 95% CI = 1.15–5.51) compared to households with a minimum of five family members.

### Disparities in modern health service utilization

The study evaluated the effects of the CBHI scheme on the reduction of disparities in health service utilization among households. Accordingly, the odds of MHS utilization among insured urban households were 14.5 times more likely to utilize MHS compared to rural households (AOR 14.98, 95% CI = 5.12–43.82). Similarly, uninsured urban households were 2.24 more likely to utilize healthcare compared to rural residents (AOR 2.24, 95% CI = 1.11–4.53).

The education level was a significant factor that contributed to the disparities in healthcare utilization among insured households. Even though the households were insured, the household heads who had no formal education and had primary education were 80% less likely to utilize healthcare compared to those household heads who attended secondary education and above (AOR = 0.20, 95% CI = 0.05–0.83). However, significant differences were not observed in healthcare utilization based on the education level among uninsured households.

The study also revealed that wealth status and family size were significant factors that contributed to disparities in healthcare utilization among uninsured households. Accordingly, those households who were relatively poor were 74% less likely to utilize healthcare compared to rich households (AOR 0.26, 95% CI = 0.10–0.65). Similarly, households with less than five family members were 3.6 times more likely to utilize healthcare (AOR = 3.57, 95% CI = 1.46–8.72). In contrast, a significant difference was not observed in healthcare utilization across the wealth status and family size differences among insured households (Table 4).

**Table 4:** The effects of the CBHI scheme on the disparity in health service utilization among households in the Gida Ayana district, Oromia Regional State, Ethiopia, 2022.

**Variables**	**Response category**	**Insurance member-ship**	**Health service utilization**		**AOR, 95% CI**	
**Yes (%)**			**No (%)**	**Insured**	**Uninsured**
Place of residence	Urban	Yes	89 (44.3)	5 (2.5)	14.98 (5.12–43.82)^***^	2.24 (1.11–4.53)^*^
		No	62 (30.8)	45 (22.4)		
	Rural	Yes	33 (27.5)	26 (21.7)	1	1
		No	23 (19.2)	38 (31.7)		
Age	20–29	Yes	30 (47.6)	6 (9.5)	2.66 (0.54–12.91)	0.68 (0.16–2.85)
		No	16 (25.4)	11 (17.5)		
	30–39	Yes	60 (45.1)	9 (6.8)	1.77 (0.42–7.49)	0.85 (0.28–2.56)
		No	32 (24.1)	32 (24.1)		
	40–49	Yes	20 (23.3)	10 (11.6)	0.61 (0.13–2.92)	1.49 (0.51–4.40)
		No	28 (32.6)	28 (32.6)		
	Above 50	Yes	12 (30.8)	6 (15.4)	1	1
		No	9 (23.1)	12 (30.8)		
Family size	< 5	Yes	45 (52.3)	6 (7)	1.09 (0.30–3.97)	3.57 (1.46–8.72)^**^
		No	27 (31.4)	8 (9.3)		
	≥5	Yes	77 (32.8)	25 (10.6)	1	1
		No	58 (24.7)	75 (31.9)		
Education level	No formal education	Yes	44 (29.9)	19 (12.9)	0.19 (0.06–0.61)^**^	0.48 (0.20–1.15)
		No	36 (24.5)	48 (32.7)		
	Primary	Yes	21 (27.6)	7 (9.2)	0.20 (0.05–0.83)^*^	0.82 (0.32–2.12)
		No	27 (35.5)	21 (27.6)		
	Secondary and above	Yes	57 (58.2)	5 (5.1)	1	1
		No	22 (22.4)	14 (14.3)		
Wealth status	Poor	Yes	97 (37.3)	26 (10)	0.87 (0.23–3.32)	0.26 (0.10–0.65)^***^
		No	62 (23.8)	75 (28.8)		
	Rich	Yes	25 (41)	5 (8.2)	1	1
		No	23 (37.7)	8 (13.1)		

^*^p-value < 0.05.

^**^p-value < 0.01.

^***^p-value < 0.001.

1, reference; CI, confidence interval; AOR, adjusted odds ratio.

## Discussion

Countries around the world are implementing different healthcare strategies to achieve the goal of UHC, which is to ensure that healthcare is accessible to all with sufficient quality and without suffering financial hardship. However, LMICs are not achieving their intended goal because of different challenges. As a major challenge, to mitigate the financial barriers to healthcare access and utilization of the service, these countries are implementing health insurance (19).

In the current study, the effect of health insurance on health service utilization among households was estimated. Thus, the overall level of health service utilization was determined to be 64.5% among general households, whereas the level of modern healthcare utilization was higher among insured, which was 79.7 and 50.6% among insured and uninsured households, respectively. This indicated that the CBHI scheme significantly improved the utilization of modern health services among households, which was in line with a study conducted in America, which showed that people with health insurance were significantly more likely to use healthcare and used care frequently; nearly 75% of those who had health insurance used outpatient care compared with approximately half of those without health insurance (20).

The study also showed that being an insurance member among households on average increased the number of modern health facility visits by 1.5 per household compared to uninsured household visits. It is similar to the study conducted in America, which showed that the median number of times a respondent used outpatient care during the year among people with health insurance was 2, while for those without insurance, it was 1 (20). Similarly, the study results in Ethiopia showed that households that were enrolled in CBHI were 50.5% more likely to utilize healthcare compared to 29.3% of non-insured (18), the study result in the Southern Ethiopia showed that the level of outpatient healthcare services utilization was 88.5% and 72.3% among insured and uninsured households respectively, and that insurance member households had a higher utilization rate of 39.1% than 25% of non-member households in public hospitals (21). Similarly, the study conducted in Saudi Arabia showed that receiving health insurance increases the chances of attending a medical check-up by ~13%−20% (22). The finding was also consistent with previous studies in India, where utilization of healthcare services was 6%−7% higher among scheme members than non-members, and in Burkina Faso, where rates of healthcare visits were 30% for insured compared to 12% for uninsured household members (23).

The study also estimated the odds of modern health service utilization among insured households compared to non-insured households. Accordingly, the study showed that the CBHI scheme has significantly improved the utilization of healthcare, and thus, insured households utilized modern healthcare 4.3 times more likely than uninsured households. Similarly, the study conducted in America showed that individuals with health insurance had 2.39 higher odds of using outpatient care than individuals who lacked insurance (20); the study in South Africa showed that households with medical insurance were 5.4 times more likely to utilize health services (24); and the study in Ethiopia showed that the insured households were almost three times more likely to utilize healthcare compared to their counterparts (21, 25).

In contrast, the study conducted in Vietnam showed that there was no significant evidence that health insurance increased the mean number of inpatient and outpatient visits at the hospitals (26). Probably, this difference could be due to the difference in the existing healthcare system, and the current study included different health facilities such as hospitals, health centers, clinics, and health posts, whereas the study conducted in Vietnam focused on health service utilization only in the hospitals.

In addition, the study also identified that being an urban resident had a significant effect on the utilization of healthcare, and thus, urban households were 4.5 times more likely to utilize modern health facilities. The study clearly showed that, whether households were insured or uninsured, significant disparities were observed in MHS utilization among urban and rural households. Even though households were insured, the odds of healthcare utilization were significantly higher among urban households compared to rural households.

Similarly, the study conducted in Greece revealed that urban populations were more likely to use primary health services compared to populations from rural areas (27), and the study conducted in Iran also showed that 58% of urban and 42% of rural populations utilized healthcare (28), and a study in Wales showed that rural households were less likely to go to modern health facilities than urban households (29). This could be because urban areas have more modern health facilities that are easily accessible, enabling urban households to seek and utilize modern health services more likely compared to rural households.

The study also revealed that household heads who had no formal education and were unable to read and write were 66% less likely to utilize modern health services compared to educated households. Even though the households were insured, significant disparities in MHS utilization were observed across the education levels of household heads.

It is similar to the study finding in Greece, which showed that primary education was associated with more health facilities visits (27). This could be because, when the household heads have formal education, they can easily understand the perceived morbidity of their family members and the health service availability in modern health facilities.

Again, the present study showed that households with less than five members were at least two times more likely to utilize MHS compared to those households with a minimum of five family members, and this was true among uninsured households. Similarly, the study conducted in Nigeria revealed that parents who had only one child were eight times more likely to seek and utilize healthcare within 24 h of the onset of illness (30). This could be due to households with more family sizes having more expenditures on other basic needs to lead their family rather than spending their income on healthcare, which could result in a delay in healthcare seeking and utilization or looking for other options for their perceived morbidity. In contrast, significant disparities were not observed in MHS utilization across family size differences among insured households, which revealed that CBHI significantly reduced disparities in healthcare utilization that could be affected by the family size of the households.

The study also estimated the effect of wealth status on the utilization of modern health services among general households. Accordingly, relatively poor households were 67% less likely to utilize MHS compared to relatively rich households. Similarly, uninsured and poor households were 74% times less likely to utilize healthcare. This is in line with the study conducted in South Gondar of Ethiopia, which showed that households with a medium wealth index were three times more likely to utilize healthcare compared to poor (9); the study conducted in Southern Ethiopia also revealed that households with high income were five times more likely to utilize healthcare compared to those households with low income (21); and the study conducted in Dessie, Ethiopia, showed that the households with high annual income were four times more likely to utilize modern health services compared to households with low income (31).

In contrast, no significant differences in healthcare utilization were observed among insured households, regardless of wealth status. This showed that the CBHI scheme significantly reduced the disparities in MHS utilization that could be affected by the wealth status of households.

Even though the study well estimated the effect of the CBHI scheme on disparities in odds of MHS utilization among households, the study could have limitations regarding the recall bias of the study, especially in recalling the frequency of health facility visits and utilization of the health services for the perceived morbidity in which the family members of the households became ill in the past 6 months.

## Conclusion

Overall, the CBHI scheme significantly improved MHS utilization and reduced disparities in healthcare utilization across wealth status and family size among insured households. However, despite the households being insured, the disparities in odds of healthcare utilization across the place of residence and education level differences among households were not significantly reduced. Hence, strengthening the CBHI scheme and focusing on the place of residence and education level of households are recommended to improve MHS utilization and reduce its disparities.

## Data availability statement

The raw data supporting the conclusions of this article will be made available by the authors, without undue reservation.

## Ethics statement

The studies involving human participants were reviewed and approved by the Ethical Clearance (Reference number: WU/RD/513/14) was obtained from the Research Ethics Review Committee (RERC minutes No: 19/2021) of Wollega University, Institute of Health Science. The patients/participants provided their written informed consent to participate in this study.

## Author contributions

EG contributed to data analysis, interpretation, report writing, and manuscript preparation and acted as the corresponding author. AW contributed to developing the data collection tools, data collection, and data entry for statistical software. BE contributed to the data development collection tool, data collection supervision, and report writing. All authors participated in developing the study concept and design of the study.
